# Chronic effects of two rutile TiO_2_ nanomaterials in human intestinal and hepatic cell lines

**DOI:** 10.1186/s12989-022-00470-1

**Published:** 2022-05-17

**Authors:** Pégah Jalili, Benjamin-Christoph Krause, Rachelle Lanceleur, Agnès Burel, Harald Jungnickel, Alfonso Lampen, Peter Laux, Andreas Luch, Valérie Fessard, Kevin Hogeveen

**Affiliations:** 1 Toxicology of Contaminants Unit, Fougères Laboratory, ANSES, French Agency for Food, Environmental and Occupational Health & Safety, 10 B rue Claude Bourgelat – Javené, 35306 Fougères, France; 2 German Federal Institute for Risk Assessment, Max-Dohrn-Straße 8-10, 10589 Berlin, Germany; 3 MRic Cell Imaging Platform, BIOSIT, University of Rennes 1, 2 avenue du Pr Léon Bernard - CS 34317, 35043 Rennes, France

**Keywords:** Nanotoxicology, Nanomaterials, TiO_2_, Repeated exposure, HepaRG, Caco-2

## Abstract

**Background:**

TiO_2_ nanomaterials (NMs) are present in a variety of food and personal hygiene products, and consumers are exposed daily to these NMs through oral exposition. While the bulk of ingested TiO_2_ NMs are eliminated rapidly in stool, a fraction is able to cross the intestinal epithelial barrier and enter systemic circulation from where NMs can be distributed to tissues, primarily liver and spleen. Daily exposure to TiO_2_ NMs, in combination with a slow rate of elimination from tissues, results in their accumulation within different tissues. Considerable evidence suggests that following oral exposure to TiO_2_ NMs, the presence of NMs in tissues is associated with a number of adverse effects, both in intestine and liver. Although numerous studies have been performed in vitro investigating the acute effects of TiO_2_ NMs in intestinal and hepatic cell models, considerably less is known about the effect of repeated exposure on these models. In this study, we investigated the cytotoxic effects of repeated exposure of relevant models of intestine and liver to two TiO_2_ NMs differing in hydrophobicity for 24 h, 1 week and 2 weeks at concentrations ranging from 0.3 to 80 µg/cm^2^. To study the persistence of these two NMs in cells, we included a 1-week recovery period following 24 h and 1-week treatments. Cellular uptake by TEM and ToF–SIMS analyses, as well as the viability and pro-inflammatory response were evaluated. Changes in the membrane composition in Caco-2 and HepaRG cells treated with TiO_2_ NMs for up to 2 weeks were also studied.

**Results:**

Despite the uptake of NM-103 and NM-104 in cells, no significant cytotoxic effects were observed in either Caco-2 or HepaRG cells treated for up to 2 weeks at NM concentrations up to 80 µg/cm^2^_._ In addition, no significant effects on IL-8 secretion were observed. However, significant changes in membrane composition were observed in both cell lines. Interestingly, while most of these phospholipid modifications were reversed following a 1-week recovery, others were not affected by the recovery period.

**Conclusion:**

These findings indicate that although no clear effects on cytotoxicity were observed following repeated exposure of differentiated Caco-2 and HepaRG cells to TiO_2_ NMs, subtle effects on membrane composition could induce potential adverse effects in the long-term.

**Supplementary Information:**

The online version contains supplementary material available at 10.1186/s12989-022-00470-1.

## Introduction

Due to their unique properties compared to their bulk form, TiO_2_ nanomaterials (NMs) have been introduced into a wide range of consumer products including food and food packaging applications [1–3]. As well, the use of the food additive E171, comprising approximately 36% nano-sized TiO_2_ is widespread in processed foods such as candies and chewing gums, personal care products including toothpastes [1, 4]. In a study by Weir et al. [4], the authors estimated that in the United States, the adult population is exposed to approximately 0.1 mg nanoscale TiO_2_/kg bw/day through oral exposition, and children can be exposed at much higher levels [4]. In addition, a report from the European Food Safety Authority (EFSA) in 2016 estimated human exposure to nano-sized TiO_2_ from food additives to range from 0.01 mg/kg bw per day in infants, adolescents, adults and the elderly, to 0.18 mg/kg bw per day in children [5]. The extensive use of TiO_2_ NMs in food and the daily exposure of consumers to these NMs, combined with uncertainties relating to their toxicity has raised concerns for both consumers and public health agencies.

Although the majority of ingested TiO_2_ NMs are eliminated rapidly in stool, a limited amount is absorbed by the intestine entering systemic circulation [6], and can distribute and accumulate in organs including the liver, spleen and intestine [7, 8]. Moreover, a substantial persistence of these NMs in these organs has been observed. In a study by Geraets et al. [9] where rats were exposed orally for 5 days followed by a 90-day recovery period, elimination of TiO_2_ NMs from organs was limited with 54–86% remaining in the organs after the recovery period. Indeed, nano-sized TiO_2_ particles present in human liver and spleen are likely a result of oral exposure [10]. This limited elimination of TiO_2_ NMs from organs, and the subsequent accumulation in tissues therefore represents significant concern in terms of long-term toxic effects considering the continuous daily exposure of humans to TiO_2_ NMs. A risk assessment concerning exposure to TiO_2_ NMs in food, supplements and toothpaste warned of possible health risks, notably in liver [11], and a recent evaluation of in vivo data on adverse effects of TiO_2_ NMs and comparison to levels found in human organs concluded that human health risks cannot be excluded [12].

Adverse effects associated with repeated exposure to TiO_2_ NMs have been demonstrated in vivo in rodent models in the intestine [13–16] and liver [13, 15, 17, 18]. In a study by Wang et al. [13], the authors reported that a 30 day oral administration of anatase TiO_2_ NMs to young and adult rats was associated with liver damage and hepatic edema and comprised intestinal permeability [13]. Intestinal effects were also observed in the modification of the composition of gut microbiota that was associated with morphological changes and inflammatory infiltration in the colon of rats treated for 30 days with TiO_2_ NMs (Chen et al. 2019). In addition, adverse effects in both the intestine [14, 16] and liver [18, 19] have been shown to be accompanied by inflammatory responses following exposure to TiO_2_ NMs.

The majority of in vitro studies on the toxicity of TiO_2_ NMs in models of the intestinal epithelium [20–22] have focused on short term treatments with relatively high concentrations of NMs, which does not accurately reflect repeated exposure scenarios encountered by consumers. However, a growing number of studies have studied longer-term effects following repeated exposure [23–28]. While most have reported no significant cytotoxic effects after repeated exposure in a number of cell lines, Guo et al. [23] observed decreased intestinal barrier function associated with increased pro-inflammatory signaling and decreased nutrient transport in a Caco-2/HT29-MTX model of the intestinal epithelium. In addition, as the liver has been reported to be a major site of accumulation of TiO_2_ NMs, a number of studies have investigated the effects of these NMs on hepatocyte cell models [20, 29–31]. In addition, an increasing number of nanotoxicological studies using more complex and multi-cellular in vitro models of the liver [32], more representative of the situation in vivo, have provided additional information about the toxicological effects of NMs following acute, prolonged and repeated exposure to TiO_2_ NMs [28, 33–35].

While NM accumulation in tissues has been observed in vivo following oral exposure, less is known about the mechanisms involved in the uptake and exocytosis, or the persistence of TiO_2_ NMs. A first step however involves the interaction of NMs with biological membranes and the subsequent internalization by endocytotic mechanisms, including macropinocytosis, which have been proposed to be involved in uptake of TiO_2_ NMs [36–38]. In addition to their role as structural components of the plasma membrane, phospholipids play a role in the process of endocytosis [39], and modifications of membrane phospholipid profiles have been reported following exposure of cells to NMs [40–42].

Internalized NMs are transferred to endosomes and following the process of endosome maturation, the majority of internalized NMs end up in lysosomes [43, 44]. Once within cells, NMs are persistent as a result of low levels of exocytosis which results in the accumulation of NMs within cells [45, 46] and tissues [9, 47]. Although the accumulation of intracellular NMs following repeated exposures to TiO_2_ NMs in cell culture models has few cytotoxic effects [24, 25], their persistence may induce subtle effects which may promote adverse outcomes in the long term [23–26].

Consumers are repeatedly exposed to TiO_2_ NMs through ingestion, and the investigation of potential toxic effects NMs on relevant organs or cell models after repeated exposure is necessary to identify the mechanisms of toxicity and clearly define the danger associated with repeated exposure. More information is needed to assess the fate of TiO_2_ following repeated ingestion. In a previous study investigating the acute toxicity of two rutile TiO_2_ NMs (NM-103 and NM-104), we did not observe any cytotoxic or genotoxic effects in differentiated Caco-2 and HepaRG cells [20]. This new study aimed at investigating the accumulation, persistence and cytotoxic effects of the same TiO_2_ NMs in the same cell models following repeated exposure. Cytotoxicity, pro-inflammatory effects, and modifications of lipid membrane composition were investigated.

## Results

### Uptake and persistence of TiO_2_ NMs in Caco-2 and HepaRG cells following repeated exposure

**Fig. 1:**
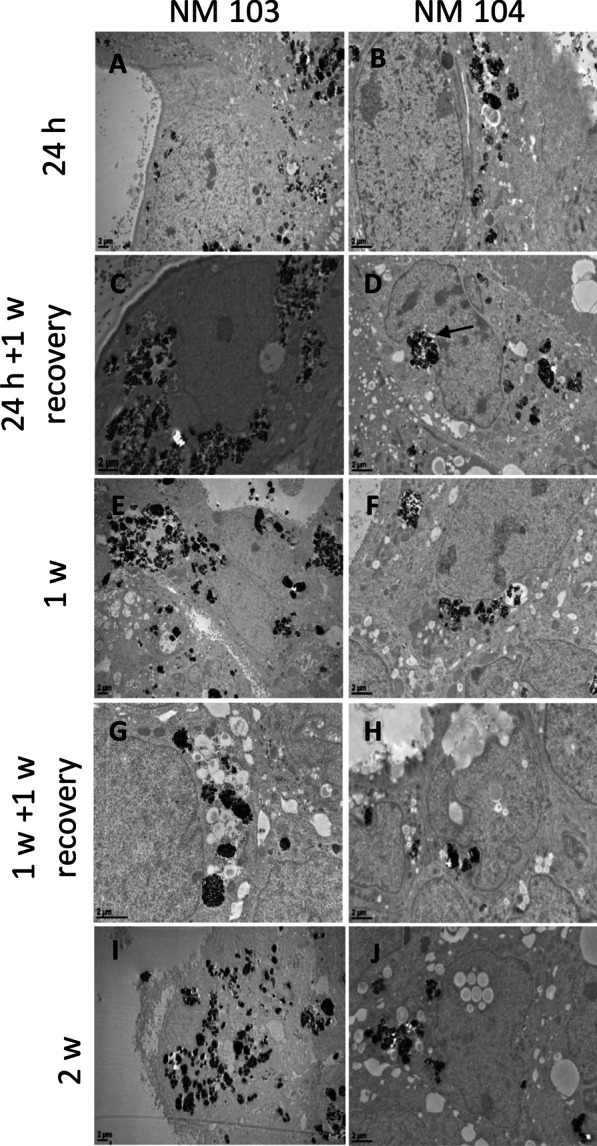
TEM images of differentiated Caco-2 cross sections showing the uptake of NM-103 (**A**, **C**, **E**, **G**, **I**) and NM-104 (**B**, **D**, **F**, **G**, **J**) exposed to 50 (**A**–**D**) or 12.5 µg/cm^2^ (**E**–**H**). NM aggregates in cell cytoplasm increase over time. Differentiated Caco-2 cells were treated for 24 h (**A**, **B**), 24 h with a 1-week recovery period (**C**, **D**), 1 week (**E**, **F**), 1 week with a 1-week recovery period (**G**, **H**), or 2 weeks (**I**, **J**). NMs were seen close to the nucleus deforming the core (full arrows)

**Fig. 2:**
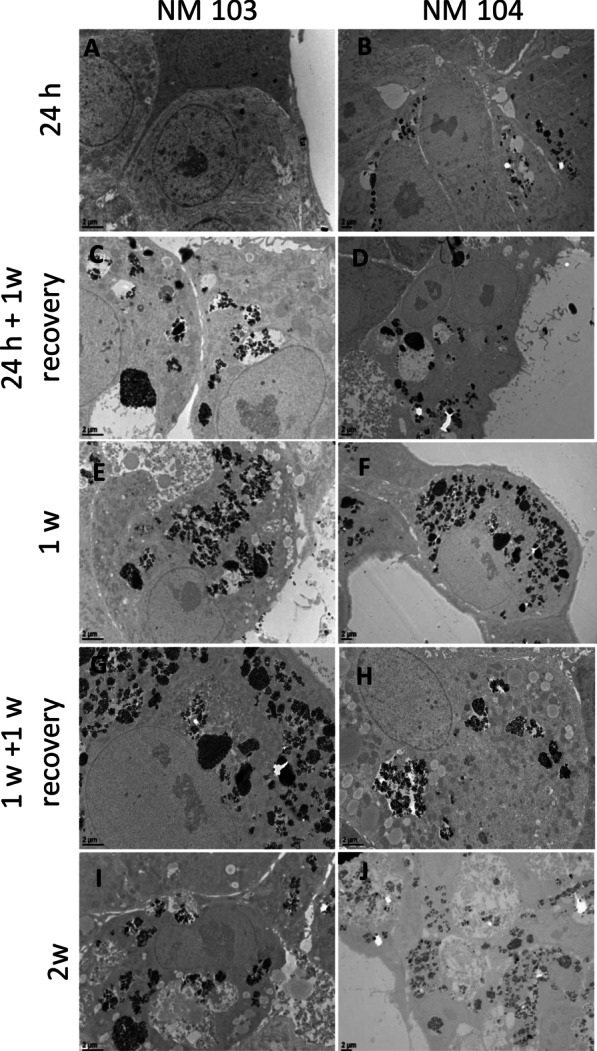
TEM images of differentiated HepaRG cross sections showing the uptake of NM-103 (**A**, **C**, **E**, **G**, **I**) and NM-104 (**B**, **D**, **F**, **G**, **J**) exposed to 50 (**A**–**D**) or 12.5 µg/cm^2^ (**E**–**H**). Differentiated HepaRG cells were treated for 24 h (**A**, **B**), 24 h with a 1-week recovery period (**C**, **D**), 1 week (**E**, **F**), 1 week with a 1-week recovery period (**G**, **H**), or 2 weeks (**I**, **J**). NM aggregates in cell cytoplasm increase over time

**Fig. 3:**
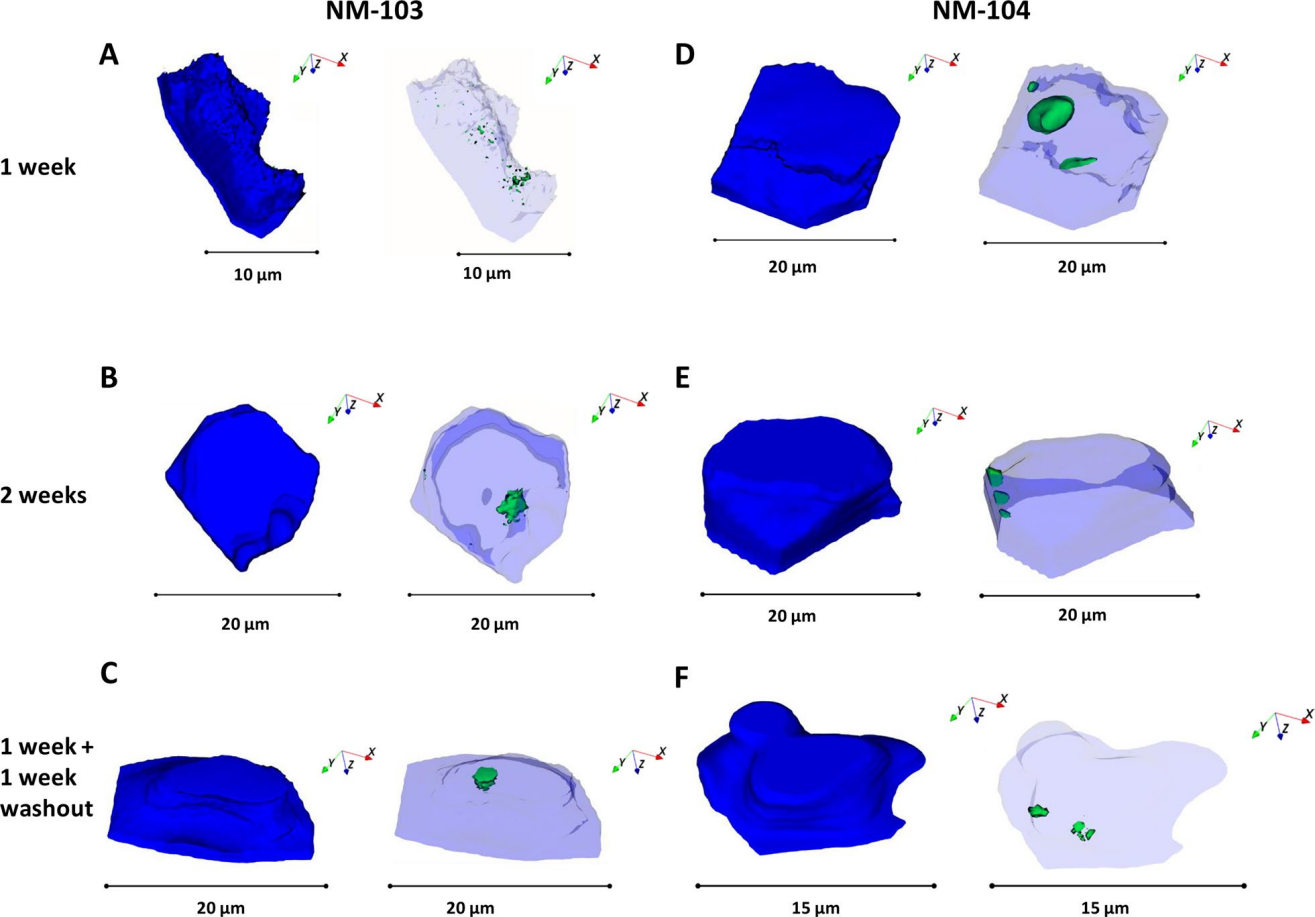
Ion reconstructions of a 3D depth profile of Caco-2 cells exposed to TiO_2_ nanoparticles at a concentration of 3.13 µg/cm^2^ (NM-103: **A**–**C**, NM-104: **D**–**F**) for 1 week (**A**, **D**), 2 weeks (**B**, **E**) and for 1 week with a 1 week recovery period (**C**, **F**). Cell outlines are shown in solid blue and were reconstructed from the C3H8N + signal from phosphatidylcholine, and intracellular TiO_2_ NMs are shown in green

**Fig. 4:**
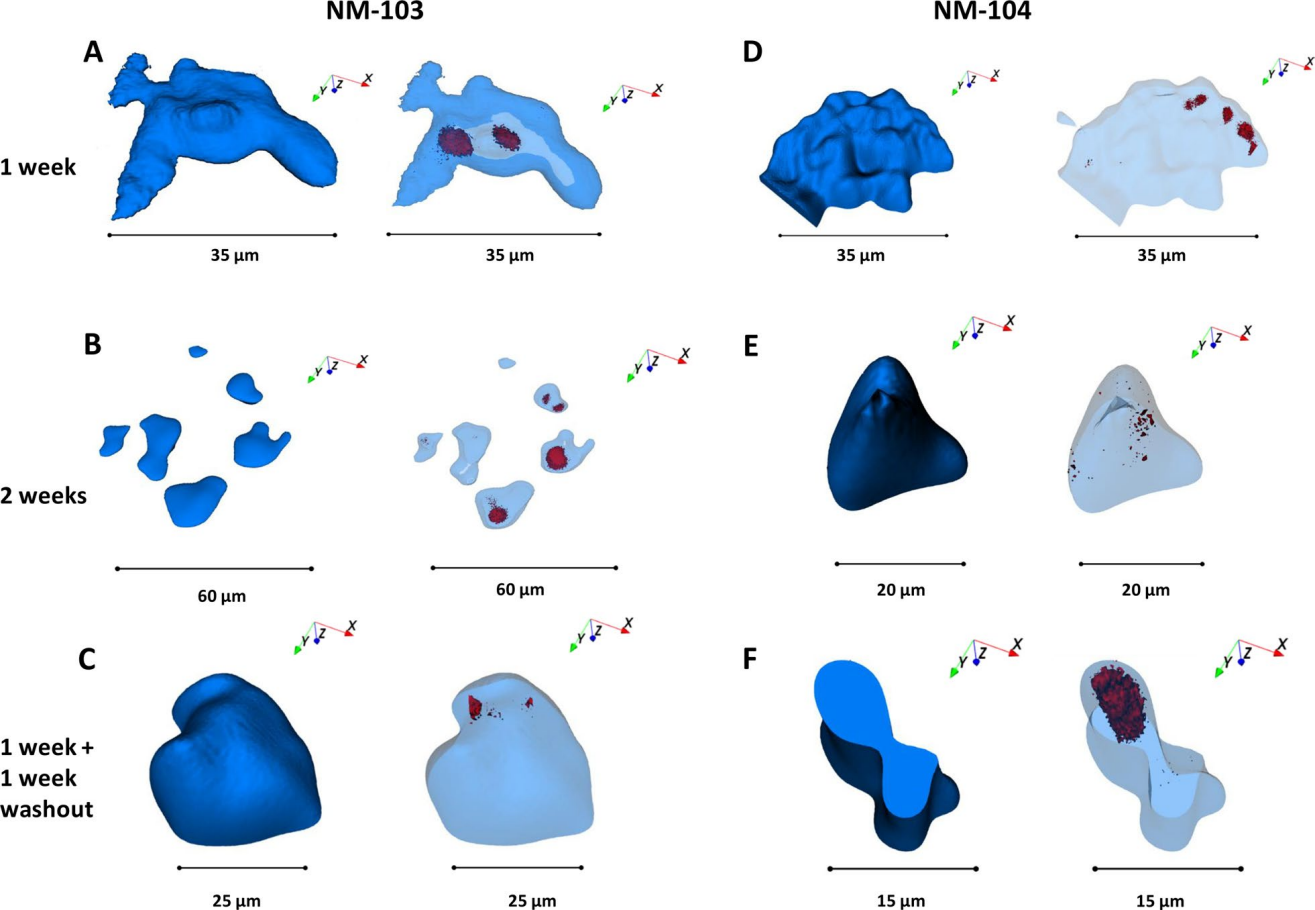
Ion reconstructions of a 3D depth profile of HepaRG cells exposed to TiO_2_ nanoparticles at a concentration of 3.13 µg/cm^2^ (NM-103: **A**–**C**, NM-104: **D**–**F**) for 1 week (**A**, **D**), 2 weeks (**B**, **E**) and for 1 week with a 1-week recovery period (**C**, **F**). Cell outlines are shown in solid blue and were reconstructed from the C3H8N + signal from phosphatidylcholine, and intracellular TiO_2_ NMs are shown in red

Uptake of TiO_2_ NMs into cells, the cellular distribution, as well as the persistence of NMs following a recovery period, was investigated by TEM in differentiated Caco-2 (Fig. 1) and HepaRG (Fig. 2) cells. Following a 24 h treatment with NM-103 and NM-104, TiO_2_ NMs were observed in the cytoplasm of differentiated Caco-2 and HepaRG cells (Figs. 1A, B, 3A, B). Although perinuclear localization of NMs was common, no particles were observed inside the nucleus of either cell line under any treatment condition. The majority of TiO_2_ NMs were present in vacuoles and endosome- and lysosome-like structures. Interestingly, in cells treated for 24 h with a 1-week recovery period without NMs, TiO_2_ NMs were still clearly present in the cytoplasm of both Caco-2 and HepaRG cells (Figs. 1C, D, 2C, D) after 1 week. ToF–SIMS analysis in Caco-2 and HepaRG cells treated with TiO_2_ NMs under chronic conditions confirmed the intracellular presence of NM-103 and NM-104 (Figs. 3, 4 respectively). Representative ToF–SIMS spectra from Caco-2 and HepaRG cells exposed to TiO_2_ NMs are shown in Additional file 2: Fig S2a and Additional file 3: Fig S2b respectively.

Significant accumulation of NM-103 and NM-104 was observed in the cytoplasm of Caco-2 and HepaRG cells after 1 week and 2 weeks of treatment (Figs. 1E–J, 2E–J). Interestingly, during long-term exposure, TiO_2_ NMs occupied a large portion of the cytoplasm of cells in all zones observed by TEM, indicating ready internalization and accumulation of these NMs. In chronic treatment conditions, NMs were sometimes observed in large, spacious autophagic vesicles following 1 week of treatment, and the presence of these autophagic vesicles increased with time in cells treated for 2 weeks. Cellular lysis was also observed following chronic treatment with TiO_2_ NMs (Additional file 1: Fig. S1). However, after recovery, some NMs were seen outside of the cells with no membrane invagination, although it is not possible to confirm their presence as a result of an exocytosis event. Despite the differences in hydrophobicity, no significant difference in cellular uptake or intracellular distribution was observed between NM-103 and NM-104 through TEM analysis in both Caco-2 and HepaRG cells. Strikingly, very large aggregates of TiO_2_ NMs were observed following 1 week of treatment, particularly in HepaRG cells (Additional file 1: Fig. S1).

In addition to nanoparticle agglomerates of 1–2 µm diameter, smaller nanoparticle agglomerates in the range between 100 nm and 1 µm are present within the same cells in both differentiated Caco-2 and HepaRG cells when measured by ToF–SIMS (Figs. 3 and 4 respectively). The results obtained with imaging mass spectrometry correlate very well with the results from TEM, indicating that indeed TiO_2_ nanoparticle agglomerates are present within intestinal Caco-2 and hepatic HepaRG cells after nanoparticle exposure. Both analytical methodologies showed that even after a recovery period of 1 week there are still TiO_2_ nanoparticles present within cells, indicating that nanoparticles taken up by Caco-2 and HepaRG cells are persistent, and therefore have the potential to cause long-term sub-toxic effects. It is also possible that TiO_2_ NMs tightly associated with cell membranes during treatments could be carried over during the recovery phase and be taken up by cells during the recovery period.

### Cytotoxicity of NM-103 and NM-104 in differentiated Caco-2 and HepaRG cells following repeated exposure

**Fig. 5:**
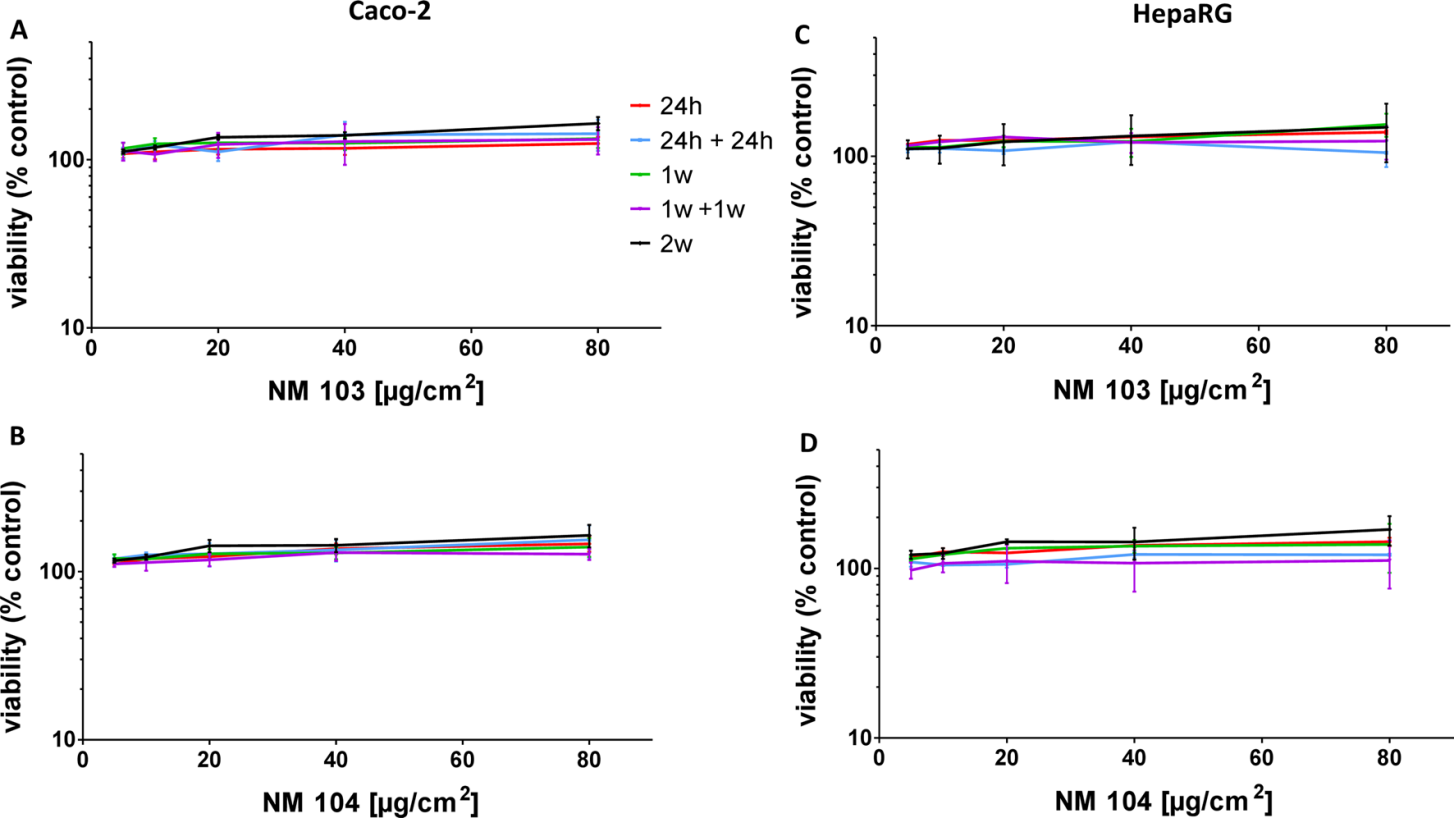
Effects of repeated treatment with TiO_2_ NMs on the viability of differentiated Caco-2 and HepaRG cells. Caco-2 cells (**A**, **B**) and HepaRG cells (**C**, **D**) were treated with concentrations of NM-103 (**A**, **C**) and NM-104 (**B**, **D**) ranging from 5 to 80 ug/cm^2^ for 24 h (light blue circles), 24 h with a 1-week recovery period (dark blue squares), 1 week (light orange triangles), 1 week with a 1-week recovery period (orange inverted triangles) or 2 weeks (black diamonds). Data are presented as the mean ± SEM from three independent experiments

Despite the presence of TiO_2_ NMs in cells, and the significant accumulation of TiO_2_ NMs in Caco-2 and HepaRG cells following repeated exposure, no significant cytotoxicity was observed by the Neutral Red Uptake assay in either cell line treated with NM-103 or NM-104 at concentrations up to 80 µg/cm^2^ under any treatment condition (Fig. 5). Although TEM images from cells treated for 1 week, 1 week + 1 week recovery, and 2 weeks displayed signs of autophagic vesicles and cellular lysis, no changes in cellular viability were observed using this assay.

### Pro-inflammatory response

**Fig. 6:**
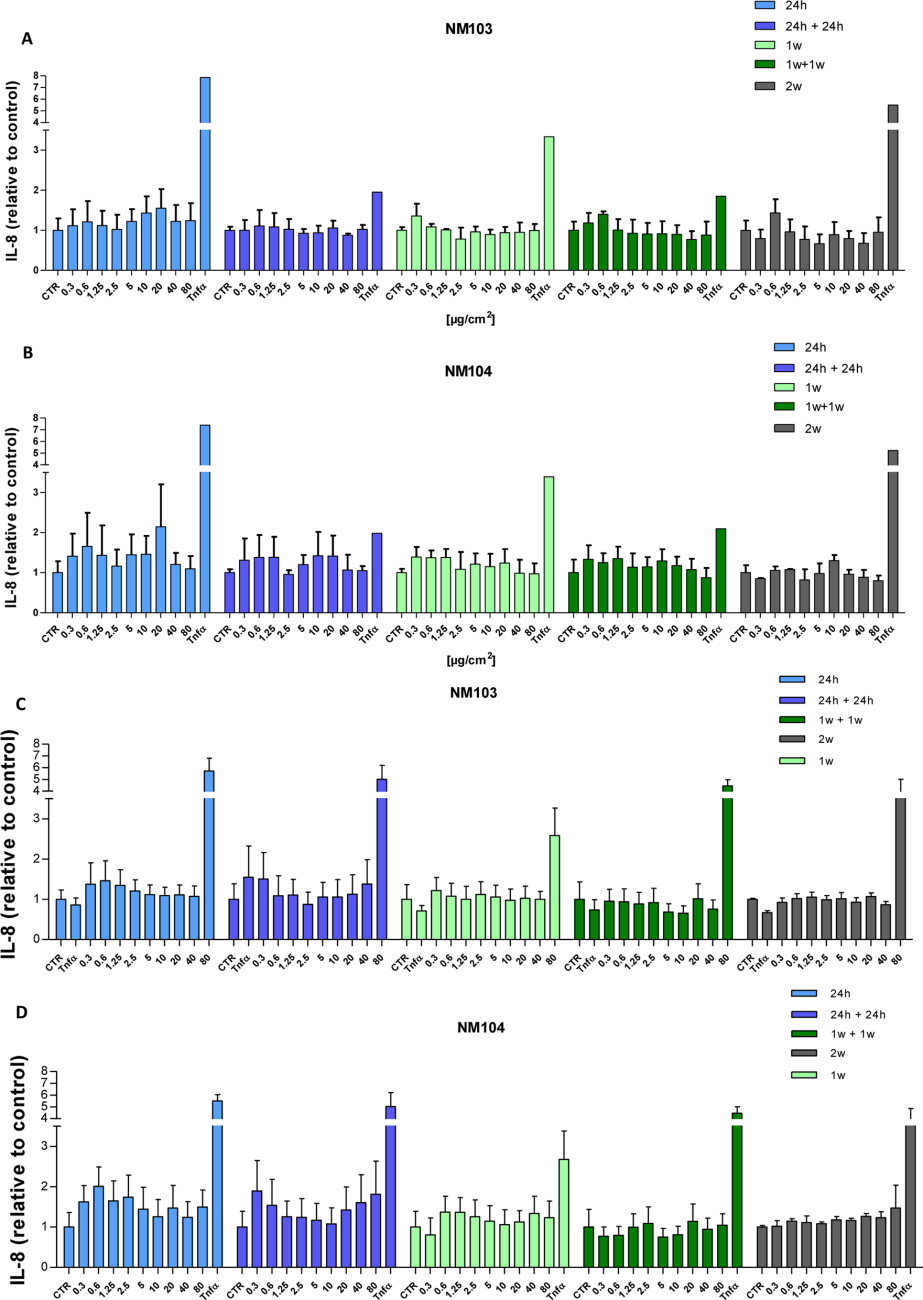
Effects of repeated treatment with TiO_2_ NMs on IL-8 secretion in differentiated Caco-2 and HepaRG cells. Caco-2 cells (**A**, **B**) and HepaRG cells (**C**, **D**) were treated with concentrations of NM-103 (**A**, **C**) and NM-104 (**B**, **D**) ranging from 0.313 to 80 ug/cm^2^ for 24 h (light blue), 24 h with a 24 h recovery period (blue), 1 week (light green), 1 week with a 1-week recovery period (green) or 2 weeks (grey). Cell culture media were collected and analyzed at the end of the treatment period. Data are presented as the mean ± SEM from three (Caco-2) or four (HepaRG) independent experiments

Under chronic treatment conditions, the pro-inflammatory response was investigated by measuring IL-8 secretion from cells exposed from 24 h to 2 weeks with or without a 1 week recovery period. Media for IL-8 ELISA assays were recuperated at the end of the treatment period, or every 2–3 days each time cells were treated. No significant change in IL-8 levels was observed at the end of 5the treatment period in either Caco-2 (Fig. 6A) or in HepaRG cells (Fig. 6B) treated with NM-103 and NM-104 at concentrations of up to 80 µg/cm^2^, nor during the course of the repeated exposure (Additional files 4, 5: Fig S3).

### Changes in membrane lipid composition

**Fig. 7:**
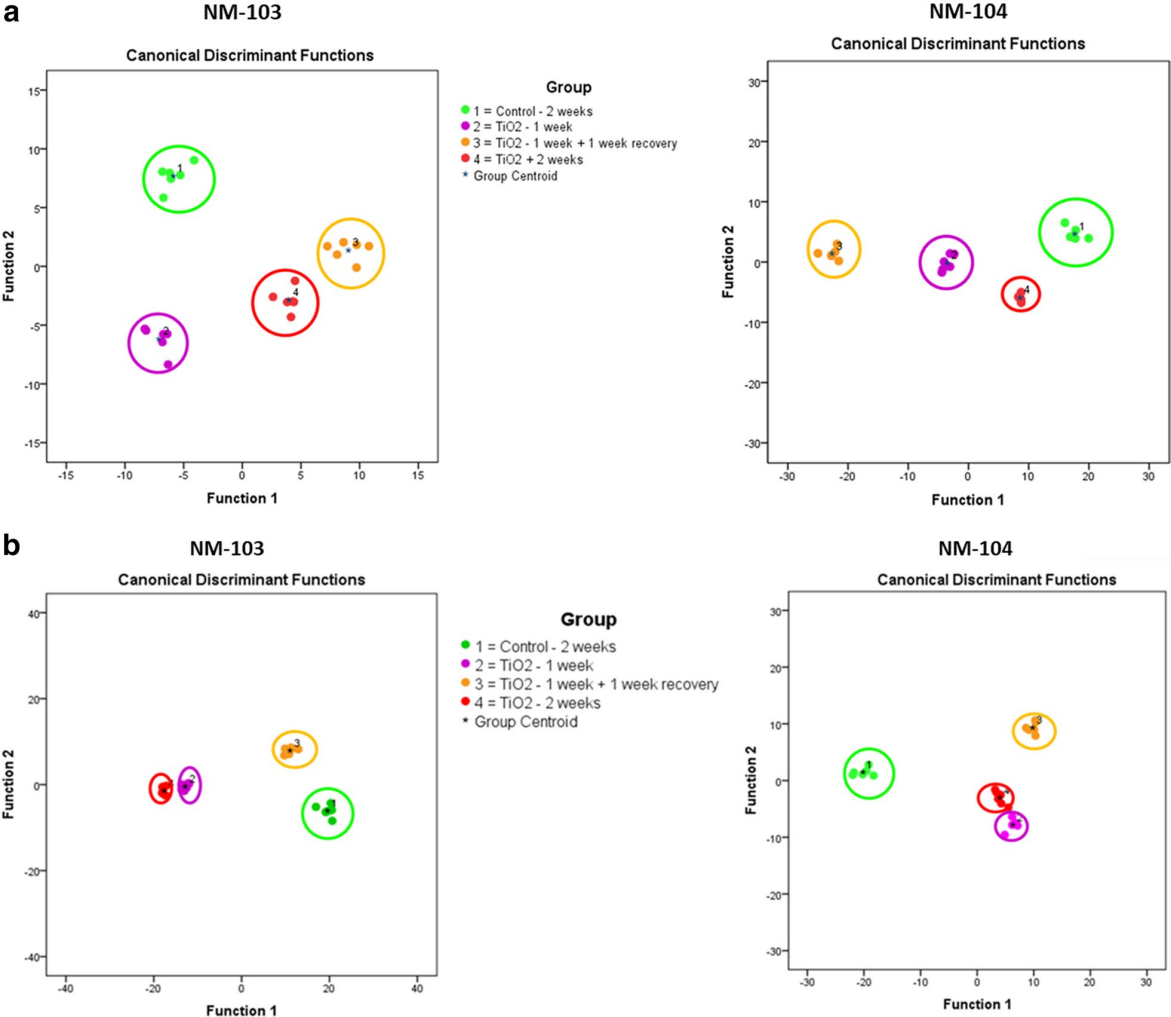
TOF–SIMS analysis of changes in compound composition of the cell membrane of Caco-2 (**A**) and HepaRG (**B**) cells after treatment with TiO_2_ nanoparticles at a concentration of 3.13 µg/cm^2^ for 1 week, 2 weeks and for 1 week with a 1-week recovery period. The diagram shows the values of the discriminant scores obtained from Fisher's discriminant analysis of 24 Caco-2 and HepaRG cell samples. The performance of the discriminant model was verified by applying the cross-validation procedure based on the “leave-one-out” cross-validation formalism (100%)

To assess whether particle uptake is correlated to changes in the lipid profile of the cell membrane in differentiated Caco-2 and HepaRG cells, we used ToF–SIMS in combination with multivariate statistical analysis [41, 42, 48, 49]. Changes in the cell membrane lipid composition for Caco-2 and HepaRG cells were observed following 1 week and 2 weeks of exposure, as well as for cells treated for 1 week with a recovery period for 1 week. Changes in membrane lipid composition were observed for both TiO_2_ nanoparticles, NM-103 and NM-104 in both Caco-2 and HepaRG cells (Fig. 7A, Caco-2) (Fig. 7B, HepaRG).

### Membrane lipid composition in Caco-2 cells following repeated exposure

**Fig. 8:**
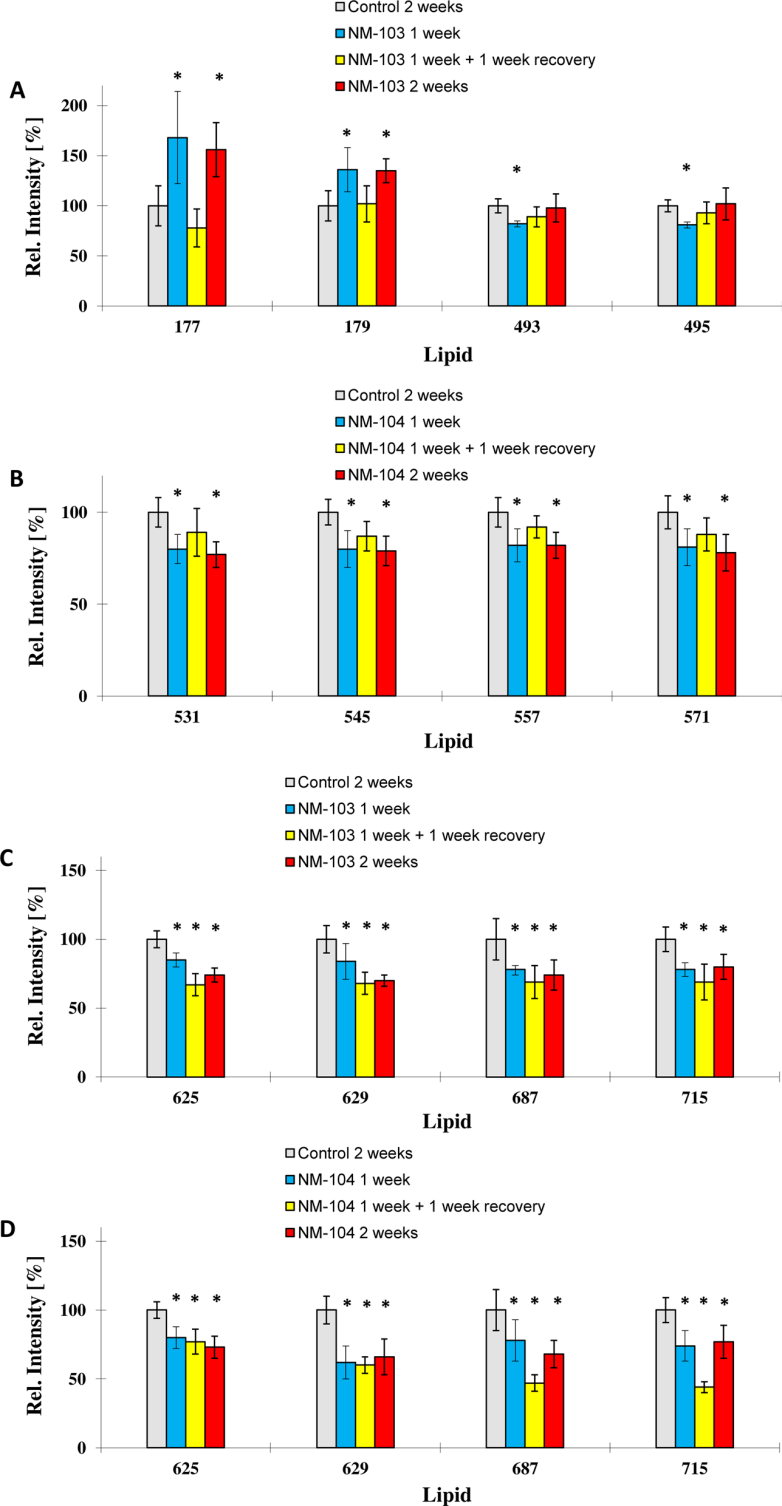
Histogram comparisons of ion yields for characteristic biomarker ions which were used to separate the four treatment groups in Caco-2 cells (unexposed control cells (2 weeks), cells exposed to TiO_2_ NMs for 2 weeks, 1 week and a washout period of 1 week). Representative biomarker ions for Caco-2 cells treated with NM-103 (**a**,** c**) and NM-104 (**b**,** d**) are presented. The biomarker ions indicate a full recovery of the cell membrane pattern to unexposed controls. For the relative intensity, the mean of the control group exposed for 2 weeks was taken as 100% in all cases

For the hydrophobic TiO_2_ NM (NM-103) at a concentration of 3.13 µg/cm^2^, ion yields of the ions m/z 177 and m/z 179 were significantly enhanced after 1 week and 2 weeks of exposure. Interestingly, cells exposed for 1 week with a 1-week recovery period had similar levels when compared to control cells that were not exposed to nanoparticles (Fig. 8A) indicating a recovery of the cell membrane pattern to control levels. Ions m/z 493 and m/z 495 significantly decreased after 1 week of exposure and recovered to control values after 2 weeks of exposure, and no difference was observed in Caco-2 cells exposed for 2 weeks compared to control cells. Ion m/z 177 was tentatively assigned asparagine, ion m/z 179 to alanine, ion m/z 493 to lyso-SM (d20:1) and ion m/z 495 to lyso-SM (d20:0).

In Caco-2 cells treated with the hydrophilic TiO_2_ NM (NM-104) at a concentration of 3.13 µg/cm^2^, ion yields for the ions m/z 531, m/z 545, m/z 557 and m/z 571 decreased significantly after 1 week and 2 weeks of exposure. Differentiated Caco-2 cells exposed for 1 week with a 1-week recovery period had similar levels of these ions compared to control cells (Fig. 8C), indicating a recovery following the recovery period. Ion m/z 531 was tentatively assigned to lyso-PG C20:5, ion m/z 545 to lyso-PI C14:0, ion m/z 557 to lyso-PG C22:6 and ion m/z 571 to lyso-PI C16:1.

Ion yields for ions m/z 625, m/z 629, m/z 687 and m/z 715 significantly decreased in Caco-2 cells treated with either NM-103 or NM-104 following 1 week and 2 weeks of exposure. Following 1 week of treatment followed by a 1-week recovery period, levels of these ions remained low, and no recovery in these ions was observed (Fig. 8D). Interestingly, m/z 687 and m/z 715 ions continued to decrease in cells treated with NM-104 for 1 week with a subsequent recovery period of 1 week.

Ion m/z 625 was tentatively assigned to lyso-PI C20:2, ion m/z 629 was tentatively assigned to lyso-PI C20:0 and ion m/z 687 was tentatively assigned to PG C30:3 and ion m/z 715 was tentatively assigned to PG C32:4.

A summary of changes in membrane lipid composition in Caco-2 cells following repeated exposure to TiO_2_ NMs is presented in Table 1.

**Table 1:** Summary of modifications in membrane composition in Caco-2 cells exposed to NM-103 and NM-104 at a concentration of 3.13 µg/cm^2^ for 1 week, 2 weeks and for 1 week with a 1-week recovery period

Caco-2		NM103		NM104	
Ion m/z	Putative identity	Effect	Recoverable	Effect	Recoverable
177	Asparagine	↑	✓	ND	ND
179	Alanine	↑	✓	ND	ND
493	Lyso-sphingomyelin (d20:1)	↑ (2 weeks)	–	ND	ND
495	Lyso-sphingomyelin (d20:0)	↑ (2 weeks)	–	ND	ND
531	Lyso-phosphatidylglycerol (C20:5)	ND	ND	↓	✓
545	Lyso-phosphatidylinositol (C14:0)	ND	ND	↓	✓
557	Lyso-phosphatidylglycerol (C22:6)	ND	ND	↓	✓
571	Lyso-phosphatidylinositol (C16:1)	ND	ND	↓	✓
625	Lyso-phosphatidylinositol (C20:2)	↓	✗	↓	✗
629	Lyso-phosphatidylinositol (C20:0)	↓	✗	↓	✗
687	Phosphatidylglycerol (C30:3)	↓	✗	↓	✗
715	phosphatidylglycerol (C32:4)	↓	✗	↓	✗

Presented ions represent ion yields for characteristic biomarker ions which were used to separate the treatment groups

### Membrane lipid composition in HepaRG cells following repeated exposure

**Fig. 9:**
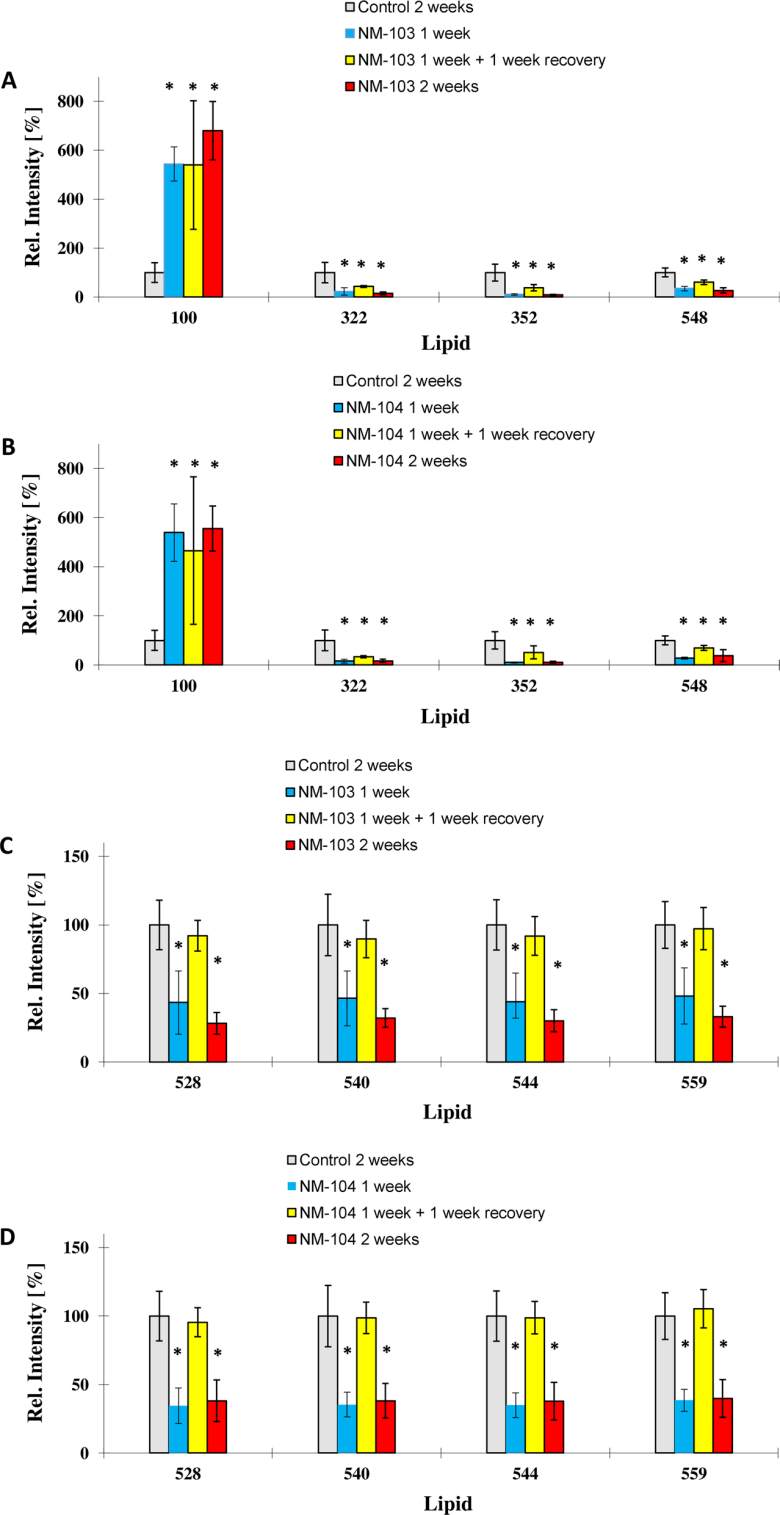
Histogram comparisons of ion yields for characteristic biomarker ions which were used to separate the four treatment groups in HepaRG cells (unexposed control cells (2 weeks), cells exposed to TiO_2_ NMs for 1 week, 2 weeks and for 1 week with a one-week washout period. Representative biomarker ions for HepaRG cells treated with NM-103 (**a**,** c**) and NM-104 (**b**,** d**) are presented. For the relative intensity, the mean of the control group exposed for 2 weeks was taken as 100% in all cases

In HepaRG cells treated with hydrophobic TiO_2_ NMs (NM-103) or hydrophilic TiO_2_ NMs (NM-104) at a concentration of 3.13 µg/cm^2^, ion yields for the ions m/z 528, m/z 540, m/z 544 and m/z 559 were significantly reduced after one and 2 weeks of exposure (Fig. 9A,B). However, these ions returned to levels comparable to those of control cells following treatment for 1 week with a 1-week recovery period indicating a recovery of the cell membrane pattern to control levels. All four ions were the highest loading ions (0.95 and higher) on PCA factor 1, which represented 76.4% of the observed variance in the model of Fig. 8 and 76.7% of the variance in the model of Fig. 9. This indicates that most cell membrane changes observed after 1 week of contact time for both nanoparticle species could be recovered to control levels and were comparable to unexposed control cells after 2 weeks.

Ion m/z 528 was tentatively assigned to lyso-PE C22:3, ion m/z 540 to lyso-PC C20:6 ion m/z 544 to lyso-PC C20:4 and ion m/z 559 to diacyl glycerol (C32:5).

A second mechanism observed resulted in a partial recovery of the cell membrane pattern for ions m/z 322 and m/z 352 (Fig. 9C,D). Ion yield for both ions was significantly reduced in 2 weeks exposed HepaRG cells, whilst the ion yield was partially recovered but still significantly lower than in unexposed control cells.

Ion yields for ion m/z 100 were significantly enhanced for both TiO_2_ nanoparticle species, NM-103 and NM-104, and could not be recovered (Fig. 9C, D). Ion m/z 100 may result from arginine.

A summary of changes in membrane lipid composition in HepaRG cells following repeated exposure to TiO_2_ NMs is presented in Table 2.

**Table 2:** Summary of modifications in membrane composition in HepaRG cells exposed to NM-103 and NM-104 at a concentration of 3.13 µg/cm^2^ for 1 week, 2 weeks and for 1 week with a 1-week recovery period

HepaRG		NM103		NM104	
Ion m/z	Putative identity	Effect	Recoverable	Effect	Recoverable
100	Arginine	↑	✗	↑	✗
322	Sphingosine-1-phosphate (d14:2)	↓	✗	↓	✗
352	Sphingosine-1-phosphate (d16:1)	↓	✗	↓	✗
548	Lyso-phosphatidylcholine (C20:2)	↓	✗	↓	✗
528	Lyso-phosphatidylethanolamine (C22:3)	↓	✓	↓	✓
540	Lyso-phosphatidylcholine (C20:6)	↓	✓	↓	✓
544	Lyso-phosphatidylcholine (C20:4)	↓	✓	↓	✓
559	Diacyl glycerol (C32:5)	↓	✓	↓	✓

Presented ions represent ion yields for characteristic biomarker ions which were used to separate the treatment groups

## Discussion

TiO_2_ NMs are currently used for a wide range of consumer applications, including their use in food products and packaging. While considerable, although often-conflicting, data on the acute in vitro toxicity of TiO_2_ NMs are currently available, very few studies have addressed chronic exposure scenarios in relevant in vitro model systems. Studies that are more representative of realistic human exposure are therefore necessary. In the present study, we have investigated the acute and repeated toxicity of TiO_2_ NMs in relevant models of target organs following oral exposure: intestine and liver. We have investigated the cytotoxicity and cellular responses of differentiated Caco-2 and HepaRG cells following repeated exposure with two rutile TiO_2_ NMs differing in hydrophobicity. This study also aimed to investigate the fate of internalized NMs during a prolonged recovery period.

The accumulation of TiO_2_ NMs in Caco-2 and HepaRG cells observed in the current study is in agreement with previous repeated-dose studies in differentiated intestinal cell models [24], although these studies were performed using different TiO_2_ NMs. Interestingly, despite a 1-week recovery period following 24 h and 1-week treatments, significant quantities of TiO_2_ NMs remain within the cytoplasm of treated cells. This indicates that TiO_2_ NMs are rapidly taken up by cells within the first 24 h, and remain within endosomal and lysosomal-like compartments for extended periods. This rapid uptake of NMs in cells is consistent with a study by Gitrowski et al. [50] that showed a rapid active uptake of TiO_2_ NMs in Caco-2 cells achieving 50% saturation after 30 min for rutile TiO_2_ NMs. Although our study does not permit a quantitative assessment of NM uptake in cells, no obvious difference in uptake of the two TiO_2_ NMs was apparent from TEM and ToF–SIMS analysis. Interestingly, a recent study using the same TiO_2_ NMs has demonstrated differential internalization of these NMs in Caco-2 cells, with NM-103 being more readily internalized when compared to NM-104 [51]. Nevertheless, the accumulation and persistence of large quantities of intracellular TiO_2_ NMs in Caco-2 and HepaRG cells during repeated exposures of up to 2 weeks suggests a remarkable ability of these cells to adapt.

The fate of TiO_2_ NMs once internalized in cells is a critical question, in particular when considering repeated exposures. Although in TEM images, TiO_2_ NMs could be observed outside of the cell in both Caco-2 and HepaRG cells, it is not possible to determine whether this is a result of exocytosis. It is interesting to note that a study performed in cycling A549 cells demonstrated only negligible export of NMs [52]. In addition, although Wang et al. [46, 53] observed that approximately 40% of TiO_2_ NMs were eliminated from neural stem cells by exocytosis after a 24 h recovery, the rate of exocytosis was negligible after 72 h [46]. It is important to note that our study used differentiated non-dividing cells whereas the former studies were performed in normally cycling cells, in which internalized NMs can be diluted during successive cell divisions [52]. Longer recovery periods could provide more information concerning the duration of this persistence of TiO_2_ NMs in Caco-2 and HepaRG cells, and could provide insight into potential adverse effects of accumulated NMs within cells. This persistence of NMs observed in vitro is also in agreement with in vivo studies showing the persistence of TiO_2_ NMs in tissues long after treatment [9]. Nevertheless, the accumulation and persistence of TiO_2_ NMs in cells indicates that nanoparticles therefore have the potential to cause long-term sub-toxic effects, perhaps long after the initial contact.

Despite the internalization and accumulation of NM-103 and NM-104 in differentiated Caco-2 and HepaRG cells, and despite observing large amounts and large aggregates of TiO_2_ NMs in cells following 1 week and 2-week treatments, no considerable cytotoxic effects were observed in either cell line. The lack of cytotoxicity demonstrated in this study is in agreement with several other studies investigating the toxicity of TiO_2_ NMs following acute [20, 22, 37], and repeated exposures in differentiated human intestinal cell models [23, 24, 54], as well as in continuously cycling cells exposed repeatedly to TiO_2_ NMs [25]. In addition, a recent study investigating the toxicity of TiO_2_ NMs in four different intestinal models of increasing complexity observed adverse effects on DNA damage only in proliferating cells, whereas no adverse effects on toxicity, DNA damage or cytokine secretion were observed in the more complex models, even following repeated treatments [27]. However, Koeneman et al. [55] reported cytotoxic effects and an increase in permeability of a Caco-2 monolayer. These cells however were treated with TiO_2_ NMs in the absence of serum, which has been shown to modify the interaction of NMs with cells, the internalization and the toxicity [56–58]. As well, anatase TiO_2_ NMs have been reported to be more cytotoxic than rutile TiO_2_ NMs [59], and the absence of cytotoxic effects observed in acute [20] or the current repeated exposure study could be related to the rutile nature of the NMs used. While the 2-week exposure in our study does not reflect the shorter lifespan of human enterocytes in vivo [60], this maximized exposure scenario does not provoke cytotoxic effects.

The accumulation of TiO_2_ NMs in the liver following oral exposure [18] has been shown to be associated with hepatic toxicity including induction of reactive oxygen species [15, 17, 61], liver inflammation [18, 61] and liver edema [13]. While several in vitro studies reported low cytotoxicity in liver cell models following acute treatment with TiO_2_ NMs [29, 30, 62], an increasing number of reports on the effects of repeated exposures on human hepatic cell models are available [28, 33, 35]. In a recent study investigating the toxicity of TiO_2_ NMs in HepG2 spheroids, no cytotoxicity was observed following acute and prolonged treatment (120 h) with these NMs [35]. However, another study in a 3D human liver microtissue model showed increased cytotoxicity of TiO_2_ NMs in prolonged and repeated exposure scenarios up to 360 h [28]. Although we cannot be certain that no TiO_2_ NM-induced cell death occurred during the repeated exposure, no significant changes in the morphology of differentiated Caco-2 or HepaRG cells were observed. However, TEM imaging of Caco-2 and HepaRG cells revealed the appearance of autophagic vesicles under chronic treatment conditions, which appeared to increase with treatment time. This appearance of autophagosomes observed in our study is common, and in agreement with several studies demonstrating the induction of autophagy in response to NM exposure [63–65], and could represent a cellular strategy to eliminate foreign material within the cell [66].

Inflammation and pro-inflammatory responses have previously been reported following both in vitro and in vivo exposure to TiO_2_ NMs [18, 61, 67–69]. In an in vivo study, Nogueira et al. [70] reported an inflammatory response increased cytokine production in the small intestine of mice treated orally for 10 days with TiO_2_ NMs. In addition, treatment with TiO_2_ NMs increased inflammation in vitro and in vivo, and worsened colitis symptoms in a mouse model of colitis [69]. In intestinal cell lines, increases in IL-8 secretion have been reported following treatment of Caco-2 cells [67, 68, 71], and a pro-inflammatory response in Caco-2 cells treated with TiO_2_ NMs was shown to be mediated by a process involving EGFR-mediated endocytosis [72]. While less is known about the inflammatory effects of TiO_2_ NMs in liver, a recent study has shown increased levels of markers associated with inflammation and fibrosis in mice following a 9 month oral treatment with TiO_2_ NMs [73]. In another study, oral administration of TiO_2_ NMs to rats resulted in hepatic injury and increases in inflammatory markers, which was ameliorated with vitamin A and vitamin E treatments [74]. In vitro, treatment of human C3A hepatoblastoma cells with rutile TiO_2_ NMs resulted in an increase in IL-8 secretion [62]. In our study, no effect on IL-8 secretion was observed at any time following repeated exposure of Caco-2 and HepaRG cells with the two rutile TiO_2_ NMs. The pro-inflammatory response may be therefore cell-type dependent. In agreement with our results, no increase in the secretion of pro-inflammatory cytokines was observed in a study on human macrophages treated with the same two TiO_2_ NMs used in our study [54].

Although in the present study no cytotoxic effects were observed in either differentiated Caco-2 or HepaRG cells following repeated exposure to two TiO_2_ NMs, we have identified changes in the lipid composition of the cell membrane. Despite the differing nature of the two TiO_2_ NMs used in our study (NM-103 hydrophobic, NM-104 hydrophilic), changes in the lipid composition in both Caco-2 and HepaRG cells were similar for both NM-103 and NM-104. Interestingly, while some of these modifications in lipid composition in both Caco-2 and HepaRG cells reverted to levels comparable to control cells following a 1-week recovery period, other modifications could not be recovered and remained at levels similar to the 1-week and 2-week treatment groups. Other studies have reported changes in lipid membrane profiles in human macrophages in response to nanosilver exposure that could be correlated to phagocytic activity and responses to oxidative stress [41, 42]. We observed increases in membrane associated arginine levels in Caco-2 and HepaRG cells treated with TiO_2_ NMs. Liver cells are known to possess an arginine uptake system, which enhances subsequent NO synthesis in hepatic cells [75], and this system seems to be activated following exposure to nanoparticles and may therefore be associated with the presence of intracellular nanoparticles. Interestingly, Kitchin et al. [76] also reported increased arginine levels and oxidative stress in HepG2 cells following treatment with CeO_2_ nanomaterials, which could be related to modulated pathways of NO synthesis. However, in a previous study investigating the acute toxicity of NM-103 and NM-104 in Caco-2 and HepaRG cells, we did not observe effects on levels of oxidative stress [20].

Interaction of NMs with biological lipid membranes may represent the first step in their internalization and accumulation within cells. TiO_2_ NMs have been shown to interact with phospholipids through both electrostatic and hydrophobic interactions [77], and these interactions can disrupt phospholipid membranes [78]. Interaction of NMs with the phospholipid membranes can induce surface reconstruction localized at positions of nanoparticle adsorption, and can thereby modify the fluidity of the membrane [53, 79] and can induce formation of holes in the membrane and removal of membrane patches [80]. A recent study has also demonstrated that TiO_2_ NMs, in addition to altering the fluidity of lipid membranes, can also increase the permeability of membranes to small molecules [81]. As well, the active uptake of NMs by endocytosis can remove regions of the cell membrane [82] and replacement may not be initiated with the same type of phospholipid, therefore overall changes of the lipid pattern of the cell membrane could occur with particle uptake. The modifications of phospholipid profiles reported in our study could therefore be associated to internalization. This is in agreement with the recovery of certain phospholipids to control levels following a 1-week recovery period.

In addition to their role as structural elements in biological membranes lysophospholipids are messenger lipids involved in the regulation of a wide range of cellular processes and signalling pathways, and are known to exert specific effects of G-protein coupled receptors and ion transporters [83]. Lysophospholipids have also been shown to inhibit Ca^2+^ ion transport and reduce mitochondrial membrane potential in rat liver mitochondria [84]. Levels of several lysophospholipids were reduced following repeated exposure to TiO_2_ NMs in both differentiated Caco-2 and HepaRG cells, which could therefore indicate impaired cellular signalling. Interestingly, a recent study has demonstrated modifications of the phosphoproteome in A549 cells exposed to TiO_2_ NMs, with several modifications associated with proteins involved in cell–cell adhesion and tight junctions [85]. Changes in membrane phospholipid composition has also been reported to be associated with impairment of cell–cell adhesion in renal cells [86]. The modification of the phospholipid profile of Caco-2 and HepaRG cells following repeated exposure to TiO_2_ NMs may therefore be consistent with the observations that exposure to TiO_2_ NMs can influence tight junction functionality and compromise the integrity of the intestinal epithelium in vitro and in vivo [23, 37, 55]. Morphological changes in microvilli, disorganization of the brush border and impairment of cell junctions were observed in vitro in a differentiated Caco-2 monolayer [55], whereas using in vivo and ex vivo murine models of the gut, Brun et al. (2014) demonstrated that TiO_2_-NPs disrupted tight junctions [37].

Modulation of membrane phospholipid composition has been reported in Caco-2 cells in response to changes in nutritional conditions [87]. The changes in phospholipid profile observed in our study could therefore be associated with the effects of TiO_2_ NMs on cellular nutrient homeostasis reported in the literature. Indeed, TiO_2_ NMs appear to modify cellular nutrient and ion homeostasis which could be linked to changes in membrane lipid composition. TiO_2_ NMs were shown to induce upregulation of a number of efflux pumps and nutrient transporters in Caco-2 cells [22]. In addition, decreased nutrient uptake was observed in Caco-2 cells exposed to TiO_2_ NMs with zinc and iron transport being decreased [23]. In another study, TiO_2_ NMs disrupted ion exchange across membranes and resulted in inhibition of the exocytosis process [88]. Disrupted ion exchange induced by TiO_2_ NMs could therefore provide an explanation for the dysregulation of elemental homeostasis and decreased Zn and Fe levels observed by Meyer et al. [51] in Caco-2 cells treated for 24 h with the same TiO_2_ NMs used in our study. Interestingly, the authors observed NM specific effects in particular on copper levels with a threefold increase with NM-103 whereas a decrease in Cu levels were observed for NM-104. The preferential uptake on NM-103 in Caco-2 cells observed by Meyer et al. could possibly explain the dyshomeostasis of copper levels with NM-103 treatment. Since lipid dysregulation [82] and metal ion dyshomeostasis [89] have been shown to be associated with a number of human diseases and metabolic disorders, repeated exposure to TiO2 NMs could induce adverse long-term effects.

## Conclusions

The daily exposure of consumers to TiO_2_ NMs through food and personal care products raises concern for their long term effects, in particular due to the persistence and accumulation of these NMs in tissues including intestine and liver. Despite the accumulation of NMs in cells, the repeated exposure to two TiO_2_ NMs differing in hydrophobicity did not generate cytotoxic effects in differentiated Caco-2 and HepaRG cells and no pro-inflammatory response was observed in either cell line. However, our study demonstrates that once internalized, NM-103 and NM-104 TiO_2_ NMs were persistent, and significant quantities remained within cells following a 1-week recovery period. While no effects on viability and inflammatory response were observed in in vitro intestinal or hepatic models, significant changes in the membrane phospholipid composition were observed in both Caco-2 and HepaRG cells. Interestingly, while most of the phospholipid changes observed following 1 week of treatment could be reversed after a 1-week recovery period, others could not be recovered. Indeed, the impact of TiO_2_ NMs on cell membranes has been rarely investigated. While these changes are subtle, they may have an impact on cellular signalling pathways, and long-term toxicological effects cannot be ruled out due to TiO_2_ bio-persistence effects. Our results also suggest that further investigation is warranted to determine the long-term toxicological effects of bio-persistent particles. In light of the importance of membrane lipids in a wide range of biological processes in health and disease, changes in membrane lipid composition following repeated exposure to TiO_2_ NMs could therefore result in potential adverse effects to consumers.

## Materials and methods

### Chemicals

Dimethylsulfoxide (DMSO) and insulin were purchased from Sigma (St. Quentin-Fallavier, France). Williams' E medium, Fetal Bovine Serum (FBS) fetalclone II for HepaRG cells, penicillin and streptomycin were purchased from Invitrogen Corporation (Illkirch, France). Hydrocortisone hemisuccinate was from Upjohn Pharmacia (Guyancourt, France). Hyclone™ DMEM/high glucose was obtained by GE Healthcare Life Science (Logan, UT) and FBS for Caco-2 cells from Capricorn scientific (Ebsdorfergrund, Germany). Primary monoclonal anti-human IL-8 antibody (M801), biotin-conjugated anti-human IL-8 (M802B), recombinant human IL-8, HRP-Conjugated Streptavidin (N100), SuperBlock blocking buffer, 3,3′,5,5′-tetramethylbenzidine (TMB), Recombinant human Tumor Necrosis Factor alpha (TNF-α) and Tween-20 were obtained from Thermofisher Scientific (Waltham, USA).

### Physicochemical characterization

The TiO_2_ NMs, NM-103 (rutile-hydrophobic-25 nm) and NM-104 (rutile-hydrophilic-25 nm) were obtained from the European Commission Joint Research Centre, Institute for Health and Consumer Protection (JRC-IHCP, Ispra, Italy). Complete characterization of the two TiO_2_ NMs is available in a report from the JRC [90], and has been also reported in several European projects such as NANOGENOTOX and NANoREG. The characterization of the hydrodynamic diameter of TiO_2_ by Dynamic Light Scattering (DLS) and NanoTracking Analysis (NTA) as well as zeta potential over time in dispersion solution and in media used for this study were investigated previously [20].

The NANOGENOTOX protocol was used for dispersion of the two TiO_2_ NMs [91, 92]. Particle powder was pre-wetted in absolute ethanol (0.5% of the final volume) in glass vials and dispersed at a concentration of 2.56 mg/mL in 0.05% BSA in ultra-pure water. Sonication was performed using a Branson ultrasonic sonicator with a 13 mm probe diameter in an ice-water bath for 16 min at 400 W.

### Cell culture

The human colorectal adenocarcinoma Caco-2 cell line was obtained from the European Collection of Authenticated Cell Cultures (ECACC 86010202). Cells were used between passages 25–38. Caco-2 cells were cultured in DMEM supplemented with 10% FBS, 50 U/ml penicillin and 50 μg/ml streptomycin and maintained at 37 °C with 5% CO_2_. Cells were seeded at 14,285 cells/cm^2^ either in 96 well plates (High content analysis, ELISA and neutral red uptake), or in 35 mm Petri dishes (TEM). Differentiated Caco-2 cells were obtained after 21 days in culture. Cell culture media was changed every 2–3 days.

HepaRG cells (passages 13–19) were cultured as previously described [93]. Cells were grown in William's E medium supplemented with 10% FBS, 100 U/ml penicillin, 100 μg/ml streptomycin, 2 mM glutamine, 5 μg/ml insulin and 50 μM hydrocortisone hemisuccinate. HepaRG cells were seeded in 96 well plates (High content analysis, ELISA and neutral red uptake), or in 35 mm Petri dishes (TEM) at a density of 26,000 cells/cm^2^. After 2 weeks in culture, 1.7% DMSO was added to the culture medium for two additional weeks in order to induced cell differentiation.

### Treatment design

**Fig. 10:**
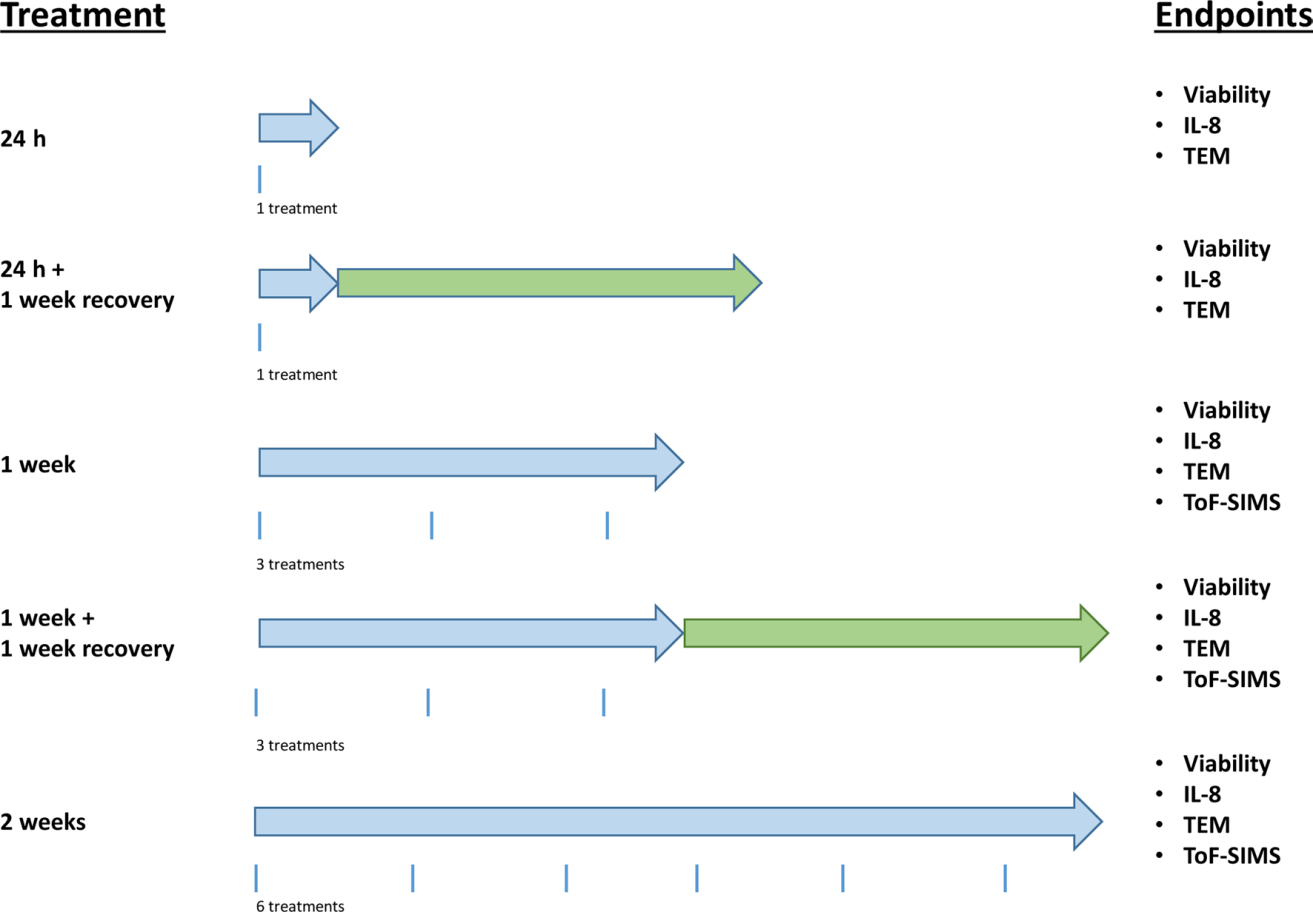
Schematic diagram of the treatment schedule for repeated exposure of differentiated Caco-2 and HepaRG cells with the two TiO_2_ NMs, NM-103 and NM-104. Blue arrows indicate exposure times, whereas green arrows indicate recovery periods in cell culture media without NMs. The various endpoints measured during each treatment condition are presented on the left

Differentiated Caco-2 and HepaRG cells were treated for 24 h, 1 week or 2 weeks with NM-103 and NM-104 at concentrations of 0.3–80 µg/cm^2^. For chronic treatments, cell culture media was replaced with fresh NM suspensions every 2 or 3 days. During a recovery period of 1 week following 24 h and 1 week treatments, cell culture media without NMs was replaced every 2 or 3 days. No wash steps were performed between treatments. A schematic diagram of the treatment protocol, including the total number of treatments in each condition is presented in Fig. 10.

### Uptake and localization in cells by transmission electron microscopy (TEM)

At the end of the treatment conditions, Caco-2 and HepaRG cells were rinsed twice with phosphate-buffered saline (PBS) and 0.15 M sodium cacodylate buffer (pH 7.2). Fixation was performed by drop-wise addition of glutaraldehyde (2.5%) for 45 min. Cells were rinsed with 0.15 M Na cacodylate buffer and post-fixed with 1% osmium tetroxide for 45 min. The samples were then rinsed with cacodylate buffer, and were dehydrated through an ethanol gradient (70–100%). Samples were infiltrated in a mixture of acetone-epon resin (50/50) for 3 h, followed by an incubation in pure epon resin for 16 h. Lastly, samples were embedded in DMP30-epon at 60 °C for 24 h. Ultra-thin Sects. (90 nm) cut using the Leica UC7 ultracut were collected onto copper grids and stained with 4% uranyl acetate, and then with lead citrate (Reynold solution). Examination of sections was performed with JEOL 1400 electron microscope operated at 120 kV equipped with a 2k–2k camera from Gatan (Orius 1000). Multiple images were analyzed from multiple zones. Images were examined by three independent observers. TEM images presented in the figures are representative of the observations from the analyzed samples. The experiment was performed two times.

### Neutral red uptake (NRU) assay

Following treatment with NM-103 and NM-104, cells were washed twice with PBS and incubated at 37 °C for 2 h with Neutral Red solution (100 μl of 33 μg/ml) prepared in PBS. After two washes with PBS, solubilization of the lysosomal neutral red was performed using 100 μl/well of NR extraction solution (1% (v/v) acetic acid and 50% (v/v) ethanol in ultra-pure water) for 5 min with agitation. The absorbances were measured with a FLUOstar Optima microplate reader (BMG Labtek, Champigny sur Marne, France) at 540 nm.

In order to account for interference with the assay due to the absorbance of TiO_2_ NMs, control wells for each nanoparticle concentration were treated as previously described, except that they were incubated with PBS in the place of Neutral Red for 2 h. Mean optical density values from treated cells incubated without NR were substracted from mean OD values from treated cells incubated with NR (ODNMs = ODcells + NMs with NR − OD cell + NMs and ODControl = ODcells control + with NR − OD cell control). Cellular viability was calculated by OD = (ODNMs/ODcontrol) × 100.

Statistical significance was evaluated using GraphPad Prism 5 with a one-way Analysis of variance (ANOVA) followed by a Dunnett's post hoc test. All error bars denote SEM. Statistical significance was depicted as follows: *p* < 0.1, *p < 0.05, **p < 0.01, ***p < 0.001.

### Time of flight–secondary ion mass spectrometry (ToF–SIMS)

Cryogenic sample preparation with a high cooling rate was used for sample analysis as previously described [42, 48, 94]. Ion images and spectra were acquired using a ToF–SIMS V instrument (ION-TOF GmbH, Münster, Germany) with a 30 keV nano-bismuth primary ion beam source (〖Bi〗x(y+)-cluster ion source with a BiMn emitter. The ion currents were 0.5 pA at 5 kHz using a Faraday cup. A pulse of 0.7 ns from the bunching system resulted in a mass resolution that usually exceeded 6000 (full width at half-maximum) at m/z < 500 in positive ion mode. The primary ion dose was controlled below 10^12^ ions/cm^2^ to ensure static SIMS conditions. Charge compensation on the sample was obtained by a pulsed electron flood gun with 20 eV electrons. The primary ion gun scanned a field of view of 500 µm × 500 μm applying a 512 × 512 pixel measurement raster. Once the primary ion gun was aligned, a ToF–SIMS mass spectrum was generated by summing the detected secondary ion intensities and plotting them against the mass channels. The data were evaluated using the Surface Lab software (ION-TOF GmbH, Münster, Germany). Six samples were analyzed for each condition. A Multivariate Analysis of Variance (MANOVA) with a Bonferroni correction was applied to assess the differences between the different groups (*p* ≤ 0.05).

### Enzyme-linked immunosorbent assay (ELISA assay)

Cultured media from control and treated Caco-2 and HepaRG cells were collected following the final treatment, and the levels of IL-8 in cell media were measured using an IL-8 ELISA assay. In another experiment (Additional files 4, 5: Fig S3), cell culture media was collected following each change of treatment media (every 2–3 days) and IL-8 levels were measured throughout the treatment period. Briefly, 96-well plates (Maxisorp, Nunc) were coated overnight at 4 °C with 1 µg/mL anti-human IL-8 monoclonal primary antibody (3IL8-H10, Fisher Scientific, Illkirch, France). Wells of the plate were saturated with SuperBlock blocking buffer (Thermo Scientific) for 1 h, and 100 µL of cell culture supernatant or recombinant human IL-8 standards were incubated for 2 h at room temperature. After three washes with PBS-Tween 20 0.05%, biotin-conjugated monoclonal anti-human IL-8 antibodies (I8-S2, Fisher Scientific, Illkirch, France) (0.1 µg/mL) were then incubated for 1 h. Plates were then washed three times and 100 µl of streptavidin-peroxydase polymer (Sigma) was added for 45 min. The chromogenic substrate TMB (100 µl) was added, and plates were incubated for 5 min at room temperature. The reaction was then stopped with 100 µL of H_2_SO_4_ (1 M). Absorbances were read at 405 nm using FLUOstar Optima microplate reader (BMG Labtek). Culture supernatants from Caco-2 and HepaRG cells treated with TNF-α (20 ng/ml) were used as positive controls. Three independent experiments were performed in triplicate.

Statistical significance was evaluated with GraphPad Prism 5 using one-way Analysis of variance (ANOVA) followed by Dunnett's post hoc tests. All error bars denote SEM. Statistical significance was depicted as follows: *p* < 0.1, *p < 0.05, **p < 0.01, ***p < 0.001.

## Supplementary Information

**Additional file 1: Figure S1.** TEM images of HepaRG cross sections showing NM-104 outside the cell after a 2-week treatment with NM-104 at a concentration of 3.13 µg/cm^2^ (A). Cell lysis in cells exposed 2 weeks to NM-104 (B) as well as a large NM-103 aggregate similar to a phagosome after 1 week of exposure at a concentration of 12.5 µg/cm^2^ (C, D) and associated with membrane invagination (full arrow).

**Additional file 2: Figure S2.** TOF–SIMS mass spectra (positive mode), showing the titanium oxide peak TiO + in red at m/e 63.87u from Caco-2 (A) and HepaRG cells (B) treated with 3.13 µg/cm^2^ TiO2 nanoparticles. i) shows the spectra for 1 week exposed to 3.13 µg/cm2; ii) shows the spectra for 1 week exposed to 3.13 µg/cm2 with a 1 week recovery period without nanoparticle contact; iii) shows the spectra for 2 weeks exposed to 3.13 µg/cm^2^;. The upper spectrum in i), ii) and iii) shows the untreated control, the middle spectrum shows cells exposed to NM-103 and the lower spectrum shows cells exposed to NM-104. The x-axis shows the molecular weight; the y-axis shows the ion intensities for the peaks.

**Additional file 3.**

**Additional file 4: Figure S3.** Effects of repeated treatment with TiO_2_ NMs on IL-8 secretion in differentiated Caco-2 and HepaRG cells throughout the treatment period. Caco-2 cells (A, B) and HepaRG cells (C, D) were treated with concentrations of NM-103 (A, C) and NM-104 (B, D) ranging from 0.313 to 80 ug/cm^2^ for 24 h to or 2 weeks. Cell culture media were collected every 2–3 days. Data are presented as the mean ± SEM from Caco-2 (n = 1) and HepaRG cells (n = 2).

**Additional file 5.**

## Data Availability

Data and materials presented in the current study are available from the authors upon request.
