# Supplementary figures and images for: Chronic effects of two rutile TiO2 nanomaterials in human intestinal and hepatic cell lines
Source: Part Fibre Toxicol. 2022 May 17;19:37. doi: 10.1186/s12989-022-00470-1 (PMC9112549; doi:10.1186/s12989-022-00470-1)

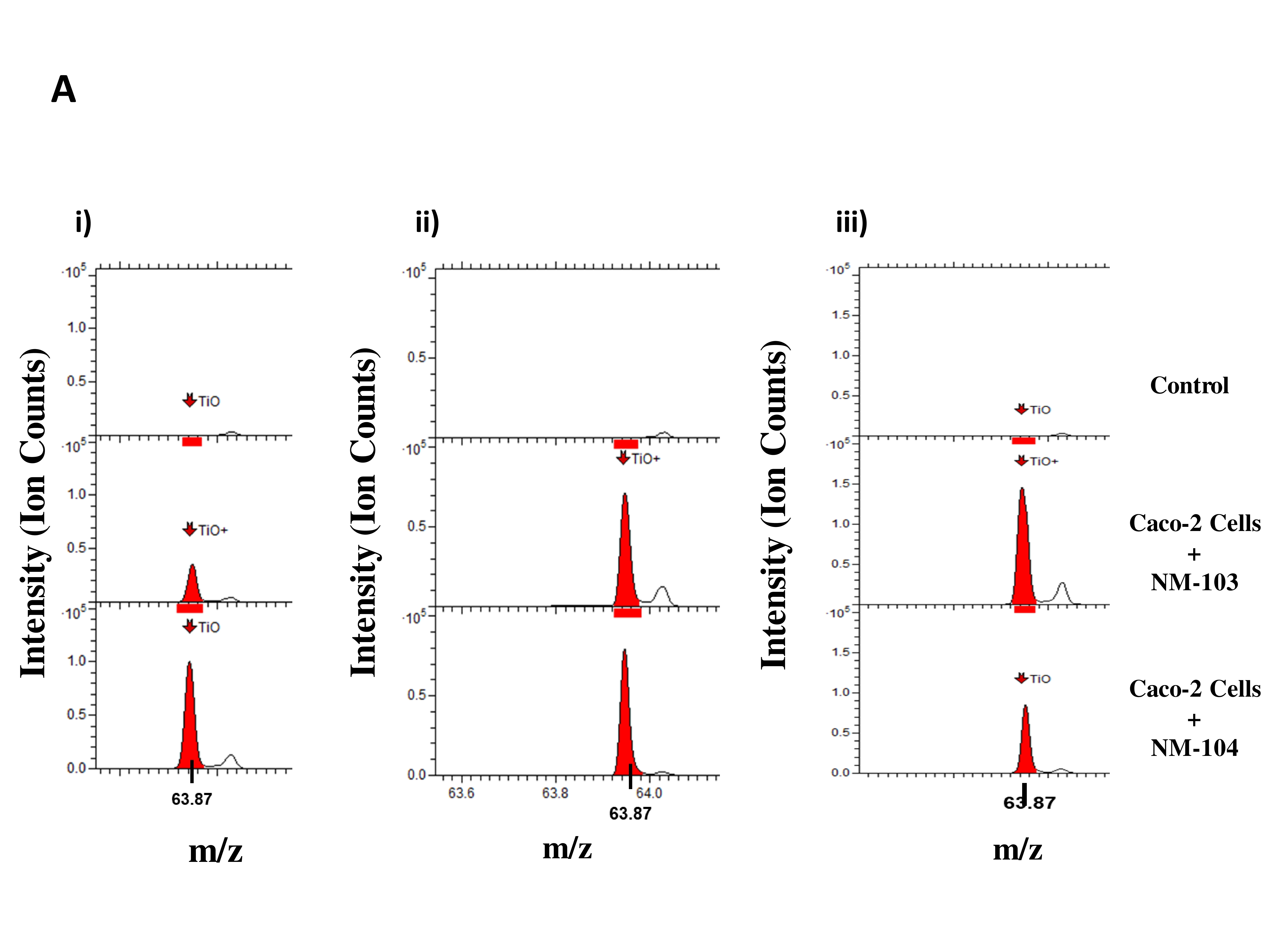

Supplement: Supplementary file 2 — Additional file 2: Figure S2. TOF–SIMS mass spectra (positive mode), showing the titanium oxide peak TiO + in red at m/e 63.87u from Caco-2 (A) and HepaRG cells (B) treated with 3.13 µg/cm2 TiO2 nanoparticles. i) shows the spectra for 1 week exposed to 3.13 µg/cm2; ii) shows the spectra for 1 week exposed to 3.13 µg/cm2 with a 1 week recovery period without nanoparticle contact; iii) shows the spectra for 2 weeks exposed to 3.13 µg/cm2;. The upper spectrum in i), ii) and iii) shows the untreated control, the middle spectrum shows cells exposed to NM-103 and the lower spectrum shows cells exposed to NM-104. The x-axis shows the molecular weight; the y-axis shows the ion intensities for the peaks. [file 12989_2022_470_MOESM2_ESM.jpg]

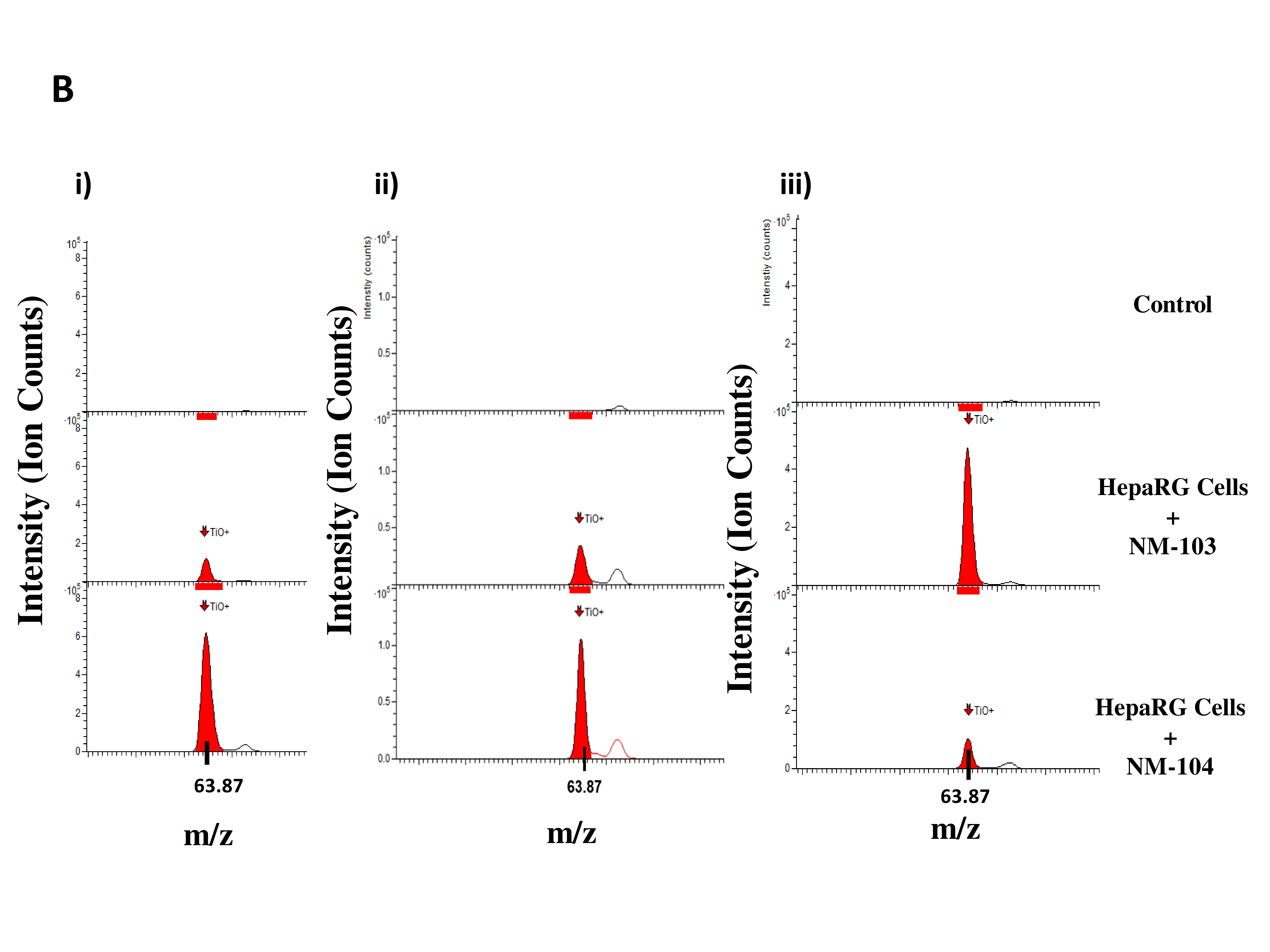

Supplement: Supplementary file 3 — Additional file 3. [file 12989_2022_470_MOESM3_ESM.jpg]

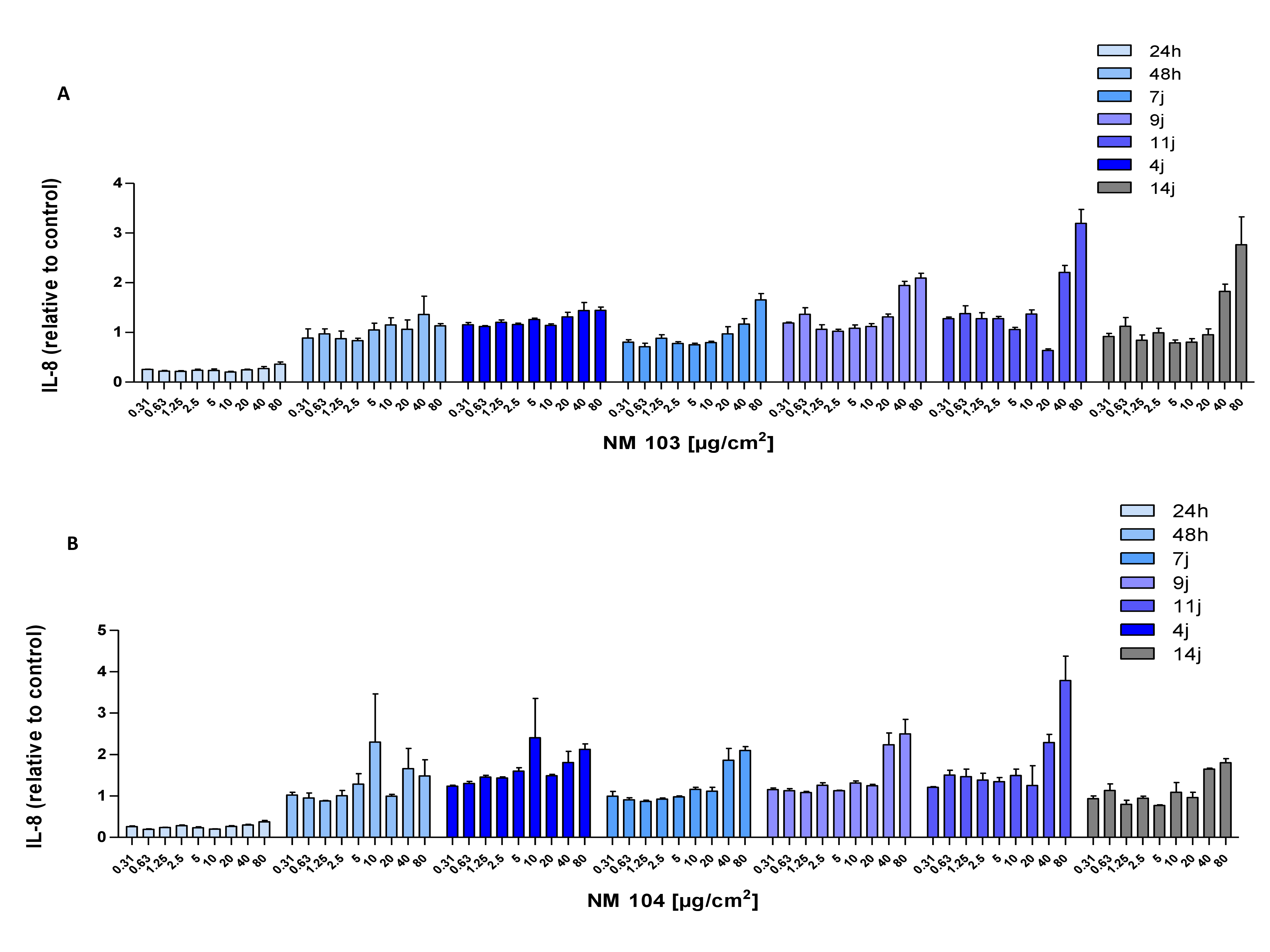

Supplement: Supplementary file 4 — Additional file 4: Figure S3. Effects of repeated treatment with TiO2 NMs on IL-8 secretion in differentiated Caco-2 and HepaRG cells throughout the treatment period. Caco-2 cells (A, B) and HepaRG cells (C, D) were treated with concentrations of NM-103 (A, C) and NM-104 (B, D) ranging from 0.313 to 80 ug/cm2 for 24 h to or 2 weeks. Cell culture media were collected every 2–3 days. Data are presented as the mean ± SEM from Caco-2 (n = 1) and HepaRG cells (n = 2). [file 12989_2022_470_MOESM4_ESM.tiff]

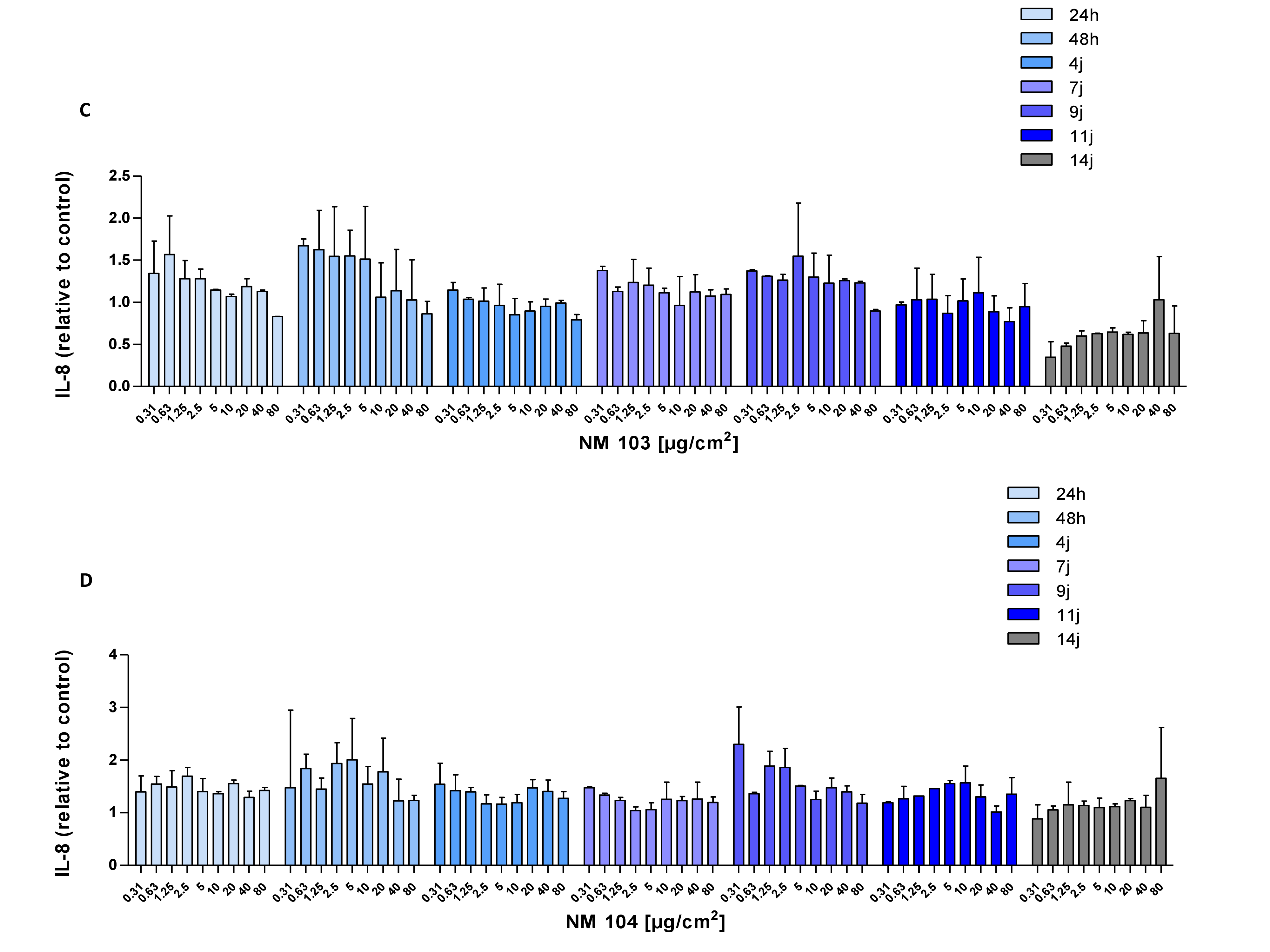

Supplement: Supplementary file 5 — Additional file 5. [file 12989_2022_470_MOESM5_ESM.tiff]
